# Implementation support practitioners – a proposal for consolidating a diverse evidence base

**DOI:** 10.1186/s12913-020-05145-1

**Published:** 2020-05-01

**Authors:** Bianca Albers, Allison Metz, Katie Burke

**Affiliations:** 1 European Implementation Collaborative, Søborg, Denmark; 2 University of Melbourne, Melbourne, Australia; 3 University of North Carolina at Chapel Hill, Chapel Hill, USA; 4 Centre for Effective Services, Dublin, Ireland

**Keywords:** Implementation support practitioner, Implementation support, Program logic

## Abstract

**Background:**

Workforce development for implementation practice has been identified as a grand challenge in health services. This is due to the embryonic nature of the existing research in this area, few available training programs and a general shortage of frontline service staff trained and prepared for practicing implementation in the field. The interest in the role of “implementation support” as a way to effectively build the implementation capacities of the human service sector has therefore increased. However, while frequently used, little is known about the skills and competencies required to effectively provide such support.

**Main body:**

To progress the debate and the research agenda on implementation support competencies, we propose the role of the “implementation support practitioner” as a concept unifying the multiple streams of research focused on e.g. consultation, facilitation, or knowledge brokering. Implementation support practitioners are professionals supporting others in implementing evidence-informed practices, policies and programs, and in sustaining and scaling evidence for population impact. They are not involved in direct service delivery or management and work closely with the leadership and staff needed to effectively deliver direct clinical, therapeutic or educational services to individuals, families and communities. They may be specialists or generalists and be located within and/or outside the delivery system they serve. To effectively support the implementation practice of others, implementation support practitioners require an ability to activate implementation-relevant knowledge, skills and attitudes, and to operationalize and apply these in the context of their support activities. In doing so, they aim to trigger both relational and behavioral outcomes. This thinking is reflected in an overarching logic outlined in this article.

**Conclusion:**

The development of implementation support practitioners as a profession necessitates improved conceptual thinking about their role and work and how they enable the uptake and integration of evidence in real world settings. This article introduces a preliminary logic conceptualizing the role of implementation support practitioners informing research in progress aimed at increasing our knowledge about implementation support and the competencies needed to provide this support.

## Background

The applied discipline of implementation science includes both implementation research and implementation practice. It aims to integrate research and practice in ways that improve the outcomes of those being served by human services [1]. Implementation research seeks to understand and evaluate the approaches that work best to translate evidence to the real world. Implementation practice seeks to apply and adapt these approaches in different contexts and settings to achieve positive outcomes [2]. For implementation practice and research to complement each other in constructive and effective ways, specific competencies are needed on both sides: Implementation researchers require knowledge and skills to conduct rigorous and relevant implementation studies; those who practice implementation - the users of the research - depend on high levels of familiarity with implementation science and the ability to select, use and tailor this science to different contexts.

The need for major research centers (e.g., the National Institutes of Health in the United States) to train researchers in dissemination and implementation research and the importance of identifying competencies for implementation research training programs has been a conversation in the field for some time [3–6]. Part of this conversation has also been the role of “*embedded researchers*” or “*researchers-in-residence*” as a potential strategy for ensuring a collaborative and participatory implementation research production that is informed by and relevant to local contexts [7–9]. While this role holds promise in bringing researchers and implementers closer together in the execution of implementation projects, its main purpose remains to carry out research [9], emphasizing the importance of *research competencies* for this role. In recent years, this emphasis on developing implementation *research* rather than *practice* competency and capacity has raised concerns about an unnecessary “wedge” between the producers and users of implementation science [10, 11], potentially creating silos in a field originally committed to their break down.

The shortage of practitioners trained in the science and practice of knowledge translation and implementation has been cited as a reason for a persistent failure to optimize the use of evidence and improve population outcomes [12]. As a result, workforce development for implementation practice has been identified as a “grand challenge” in human services [1, 13]. This has increased the scholarly interest in systematically reviewing evidence-based practice capacity building [14–16].

Findings from these systematic reviews suggest multiple core competencies needed by practitioners to adopt and implement evidenced interventions, together with strategies helpful in teaching and building such competencies. However, due to the limitations of the studies included in the reviews, these findings cannot be stratified by different audiences: Knowledge brokers as well as knowledge users, frontline staff as well as staff in formal leadership roles were considered “practitioners”. Systematic reviews that *are* focused on particular implementer roles [13, 17] aim to create a broad understanding of these roles and their contribution to evidence use but do not explore questions of competencies and capacity building. This reflects the still embryonic nature of implementation workforce development research.

In human service practice on the other hand, educational institutions increasingly offer programs to upskill students of health disciplines in implementation. However, these programs do not serve the need for practical skill development of workers, preparing them to practice implementation in the field. This signals the need to pay greater attention to particular implementation support roles and the competencies needed to pursue them. Therefore, this article aims to set the stage for a broader debate about one particular type of implementer: The “*implementation support practitioner*”.

## Main text

### Implementation support practitioners defined

Currently, a wide range of terminology is used to describe different implementation support roles. *Facilitator*, *knowledge broker*, *coach*, *consultant*, or *technical assistance provider* are among the common labels found in the literature, indicating that multiple streams of research and practice co-exist, focused on related, and potentially overlapping concepts and models.

Facilitators – sometimes also labelled “practice facilitators” – have been widely acknowledged in healthcare as a role aiming to make “*things easier for others by providing support to help them change their ways of thinking and working*” ([17] p1). Facilitators have been described as using “*a range of organizational development, project management, quality improvement, and practice improvement approaches and methods to build the internal capacity of a practice to help it engage in improvement activities over time*” [18]. However, the evidence on the effectiveness of utilizing the implementation support of facilitators remains scarce and equivocal. In a recent European, multi-site study [19] that was based on a cluster randomized design, no significant differences could be found in the compliance with continence recommendations (the primary outcome) across three study arms, two of which received different types of facilitation and one functioning as the control condition. Systematic reviews on the other hand have suggested that facilitation may help to improve different health outcomes, e.g. within chronic disease care [20] and primary care [21, 22].

The situation is similar for knowledge brokers, who have been described as primarily connecting “*researchers and decision-makers, facilitating their interaction so that they are better able to understand each other’s goals and professional culture, influence each other’s work, forge new partnerships and use research-based evidence*” [23]. While this definition is focused on the linking function of knowledge brokers, others have provided a broader description of this role, including the “*creation or synthesis, translation, dissemination, implementation, and adoption of evidence to change practice*” ([24] p223), thereby expanding the scope of knowledge brokering to active implementation support functions. The authors of a systematic review examining the role of knowledge brokers in paediatric rehabilitation, concluded that there is a limited understanding of this role – among others due to the few studies that could be identified (*N* = 4) and the diverse frameworks used to anchor the work of knowledge brokers [25]. A slightly higher number of studies (*N* = 29) could be included in a systematic review focused on knowledge brokers working in any health-related setting [13]. While this enabled the characterization of the knowledge broker as “*knowledge manager*”, “*linking agent*” and “*capacity builder*”, an assessment of the effectiveness of this role could only be built on two of the included studies with findings being inconclusive.

The role of coaches and consultants as professionals supporting other professionals in their practice is less prominent in healthcare. While “*quality improvement coaches*” have been used in single recent studies [26–28], the terms “*coaching*” and “*consultation*” are otherwise primarily linked to patient-centred approaches to improving health outcomes [29] in the form of e.g. health and life coaching [30–32], decision coaching [33] or diagnosis-specific consultations [34]. However, in mental health and education, coaches and consultants are frequently utilised to offer “*ongoing support … from a specialist to improve EBI* [Evidence-Based Intervention] *implementation after training*” ([35] p2). This support is typically offered to frontline service providers in the form of in vivo observation, practice feedback, roleplay and other strategies aimed at improving the quality with which programs are delivered to clients, students or patients [36–41]. As for other roles, the evidence on the effectiveness of coaches or consultants is limited and ambiguous. This is due to the still novel character of coaching and consultation as implementation strategies, leading to a large number of conceptual studies. These aim to unpack what is viewed as a black box of coaching or consultation and to detail these strategies and the characteristics of the individuals utilising them [35, 41, 42]. Moreover, the few empirical studies that examine the impact of coaching and consultation focus primarily on provider behaviour [38], with those examining recipient outcomes only slowly emerging [43, 44].

Finally, technical assistance (TA) has been defined as involving “*information sharing, expertise, instruction, training, and other supports for improving program, organization, or system capacity to achieve specific goals, objectives or outcomes*” ([45] p109). This definition reflects a high degree of similarity between the role of TA providers and that of the implementation support roles presented above. In healthcare, the use of the term TA at times is restricted to the implementation of specific techniques such as electronic health records or particular billing or data systems [46]. It can also be found to depict a broader type of personal support aimed at improving healthcare practice in general [47]. As part of a systematic review of the effectiveness of system-level interventions in improving the delivery of and outcomes from HIV/AIDS prevention services, technical assistance was characterized as a “*promising*” strategy based on a narrative synthesis of nine included studies that described TA as being part of the intervention [48]. A more recent review of the literature about TA provided in prevention – including 111 public, behavioural and mental health studies [49] – points to consistent weaknesses in the ways in which TA is offered, including the absence of a conceptual model informing the TA provision, and a great diversity in tasks described as core to the TA offering. The authors also highlight that the current knowledge about TA primarily stems from adoption and implementation studies and to a much lesser degree from sustainment trials.

Taken together, this shows that existing labels for implementation support roles are “*not well defined nor rigorously applied in the research literature”* ([46] p3), and that the evidence base for these roles is only emerging. To both consolidate and progress the debate and research agenda in this particular area of implementation science, we therefore suggest unifying its rather diverse terminology – under the label “implementation support practitioner” – and to develop a program logic that both utilizes past and informs future research into this role.

We suggest conceptualizing implementation support practitioners as professionals not involved in direct service delivery or management. Instead, they work closely with the leadership and staff needed to effectively deliver direct clinical, therapeutic or educational services to individuals, families and communities and support them in implementing evidence-informed practices, policies and programs, and in sustaining and scaling evidence for population impact.

A key focus of the work of implementation support practitioners is to build *provider implementation capacity,* the ability to select and apply appropriate and contextually informed implementation frameworks, strategies or other concepts and tools and to tailor these to different interventions, contexts, populations, and settings in real world health services. The educational and professional backgrounds of implementation support practitioners therefore vary. They include specialists such as clinical psychologists, social workers or nurses and generalists trained in sociology, organizational change and other fields. What connects these professionals is their knowledge and experience in supporting implementation efforts in service systems to achieve outcomes. We therefore refer to them as implementation support practitioners.

### Locating implementation support practitioners

An explicit recognition of the need to actively build the capacities of evidence users and their organizations first occurred with the development of the ‘Interactive Systems Framework’ [50], which describes evidence implementation as an interactive process unfolding between an *evidence synthesis and translation system* and a *delivery system*, assisted by a *support system*. This support system specializes in providing implementation support and building general and intervention-specific capacity to adopt and integrate research evidence into day-to-day practice. It is this support system that implementation support practitioners are part of.

Multiple studies report on how the idea of a support system can be operationalized in routine service settings [51–53]. In many cases, its operationalization has centered around an *externally situated* intermediary organization established for the explicit purpose of providing technical assistance to service providers implementing specific evidence-informed programs or interventions commissioned by government organizations [54–57]. Simultaneously, support systems have been built *within* and *between* provider organizations – in the form of distinct individual or team roles with knowledge translation and implementation support responsibilities. Examples include practice facilitators used in health [20, 21], or change agents [58–60] and implementation teams [61–63] used in child welfare services.

These examples illustrate that a support system can lie within or outside of the delivery system, with implementation support practitioners being situated within the delivery system, outside, or both. They also highlight that implementation support practitioners can and should move flexibly across the different systems that form the Interactive Systems Framework, thereby ensuring that knowledge from one system is transported to and meaningfully applied in another system.

To do this work, implementation support practitioners require specific competencies.

### A model for determining implementation support practitioner competencies

The literature on competence and competency in relation to different human service disciplines is vast and provides a multitude of definitions [64]. While there is no single, shared understanding of what competencies are, most authors agree on viewing competency as being “*more than a set of skills*” and instead a “*mix of aptitudes, attitudes and personal attributes*” ([64] p151). This understanding is reflected in a definition describing competency as “*the capability to choose and use an integrated combination of knowledge, skills and attitudes with the intention to develop a task in a certain context*” ([65] p2). This definition guides the program of work presented here. It emphasizes that a key characteristic of ‘competency’ is the ability to activate knowledge, skills and attitudes and convert them into effective ‘action’ and observable behavior [66].

In the context of implementation, this implies that implementation support practitioners need to be able to activate implementation-relevant knowledge, skills and attitudes, and to operationalize and apply these in the context of their support activities in ways that build the capacities and promote competencies in others. To determine the range of competencies they require it is therefore necessary to both identify the basic components of these competencies – knowledge, skills and attitudes – and the both personal and contextual factors that contribute to these components being combined and translated into a support practice that makes a positive difference for individuals and organizations. This thinking is reflected in an overarching logic included in Table 1.

**Table 1:**
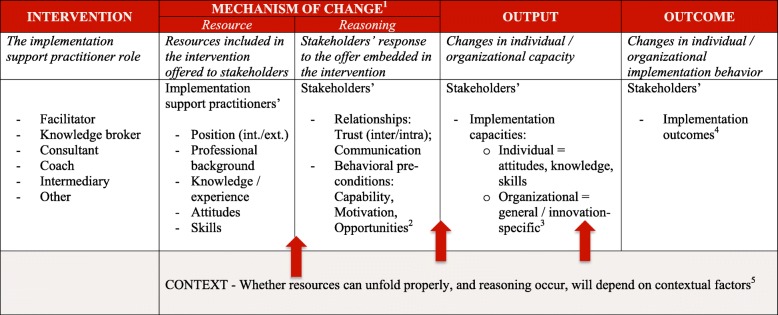
A preliminary logic for implementation support practitioners

^1^Dalkin et al. (2015) [67]

^2^Michie et al. (2011) [68]

^3^Flaspohler et al. (2008) [69]

^4^Proctor et al. (2010) [70]

^5^Damschroder et al. (2009) [71]

In outlining this logic, it is crucial to capture the potential mechanisms of change that connect the competencies of implementation support practitioners with the relational and behavior changes in the individuals and organizations they support – thereby explaining why and how such a change might occur. Rooted in scientific realism, mechanisms of change have been characterized as generating outcomes but also being unobservable, context-sensitive [72], and requiring considerable effort to operationalize in program theory development [73–75]. It is therefore not surprising that only few models aiming to capture the essence of different forms of implementation support include such mechanisms [40, 60, 76] – reflecting the still limited theoretical and empirical understanding present in the field.

In a refined approach to operationalizing mechanisms of change, scholars [67] suggest disaggregating a mechanism of change into its constituents, namely ‘resources’ and ‘reasoning’. ‘Resources’ describe the intervention that is introduced in a particular context to enable change. ‘Reasoning’ is the response from participants to the intervention, ultimately enabling change.

Using this realist approach, we view an implementation support practitioner – who may be a facilitator, knowledge broker, consultant or some other similar role – as a resource with a unique mix of qualities, including

- The formal position – implementation support practitioners can be fully embedded into the organizations and systems they support; they can be part of an intermediary organization; or they can belong to academic research settings. At times projects and initiatives may also rely on the parallel provision of services from internal as well as external implementation support practitioners. This “*blended approach*” has been frequently used in the work of the United States (U.S.) Department of Veterans Affairs [77–79].
- Professional background – i.e. training in specialist disciplines such as psychology, nursing or psychiatry or generalist education in e.g., ethnography or sociology. For example, a review of practice facilitators operating in healthcare showed that in most cases facilitators were individuals with an educational background in healthcare [22].
- Knowledge – i.e. the factual information implementation support practitioners bring to and acquire about the contexts in which they work, e.g. about the evidence-based practice or policy in focus, individual and organizational change processes or implementation concepts. As an example, in a realist review of studies examining characteristics of effective change agents operating in healthcare both practice, academic and local knowledge together with practical experience was highlighted as crucial for successful knowledge translation [80].
- Attitudes – the predispositions that influence implementation support practitioners’ work-related actions and responses, e.g. a positive attitude towards evidence-based practice, a collaborative mindset or flexibility. Among the key characteristics of effective facilitators identified through a recent systematic review were for example *self-awareness*, *self-management* and *social awareness* [81].
- Skills – the ability to activate attitudes, utilize resources and apply knowledge in ways conducive to achieving goals, e.g. the ability to engender trusting relationships, facilitate interpersonal processes or select and utilize relevant implementation strategies. For example, “*goal setting”, “assessing progress and outcomes”,* and “*providing tools and resources*” [82] emerged as the three implementation strategies that showed to be most effective in a systematic review of 35 studies focused on the role of change facilitators operating in healthcare settings [82].

We anticipate that when this resource – a unique combination of the above qualities represented in a single or multiple implementation support practitioners – unfolds as intended and required, implementation stakeholders – be it individuals, groups or entire systems – will respond positively.

In the first instance, this response will primarily be demonstrated by *trust*, which we view as foundational for implementation support practitioners to be successful. Two types of trust are important [83]. *Intrapersonal trust* is represented by the belief that the implementation support practitioner is reliable, competent, and committed to the change effort on behalf of the organization they are supporting. *Interpersonal trust* is represented by the perception of both implementation support practitioners and their stakeholders that they are in a collaborative and reciprocal relationship focused on achieving identical aims. Hence, trust describes a quality of relationships in implementation partnerships that affects information exchange and opportunities for learning [84]. While strained relationships limit the diffusion of unfamiliar and complex information, such as research evidence for implementation strategies [85, 86], trusting relationships enable individuals to engage in the risk taking, learning and behavior change required in implementation efforts.

Taking on board the concepts from the Capability-Opportunity-Motivation-Behavior (COM-B) system [68], we also suggest that relational outcomes such as trust put stakeholders in a position to enhance their implementation capability, opportunity and motivation, ultimately enabling concrete behavior change at the individual and the group / system level. *Capability* – the psychological and physical capacity to initiate behavior change – may show in changed stakeholder intentions to use research evidence, support inquiry driven service improvement, or implement an evidence-informed intervention; *motivation* in improved attitudes towards using evidenced implementation concepts; and *opportunity* – factors enabling or prompting an intended implementation behavior – in changes to the organizational climate surrounding an implementation effort.

We suggest that the consequence, or output, of this process of “reasoning” (i.e. stakeholders’ response to working with an implementation support practitioner) is two-fold. In the first instance, it will result in enhanced stakeholder implementation capacities. At the individual level, this will be demonstrated by an increase in implementation knowledge, skill and competency. Organizations and systems, on the other hand, should experience increased *general capacities* – e.g. improved organizational structures, implementation leadership, infrastructure for continuous improvement, or more evidence-focused policies – and *innovation-specific capacities*, related to the intervention in focus of the implementation support practitioners’ assistance [69]. Ultimately, the output (increased implementation capacity among stakeholders) will contribute to positive implementation outcomes measurable in the form of, e.g. greater acceptability and appropriateness of an intervention, improved fidelity assessments or an extended penetration rate – linking to the Outcomes for Implementation Research framework [70] and its inherent pathway towards better service and client outcomes.

Finally, successful implementation also requires enabling contexts, i.e. constructive adaptive system behavior [87] that supports the intended change process. Viewed from a realist perspective, the effectiveness of the processes occurring between implementation support practitioners and their stakeholders will be impacted by the influences exerted by the dynamic environments that surround and influence them.

### Steps towards building the knowledge base about implementation support practitioners

Recent literature illustrates that implementation support is frequently institutionalized in complex human services systems [14, 54, 55]. It also tells us that most technical assistance is provided without the benefit of an organizing framework or conceptual model [49, 88]. This leaves many questions unanswered about the role, work and competencies of implementation support practitioners, potentially resulting in duplication of effort, misuse of resources and negative impact on implementation science and practice.

A more unified understanding of implementation support practitioners’ role could be achieved by specifying what these actors do, the skills they need to execute their roles, and the ways in which the stakeholders they assist might respond to their offering. This should permit better analysis of implementation support practitioners’ impact on implementation processes and outcomes. It should also address recent calls for a classification system specifying the implementation actor and the implementation target to examine how specific strategies delivered by certain actors can facilitate an effective use of evidence in practice [89].

The proposed logic presented here is of preliminary nature and requires critical review and refinement. This will be part of an ongoing program of work by the authors, involving two parallel activities:

1. An analysis of data gathered from an international sample of implementation support practitioners, reflecting on the competencies they require in their day-to-day work to support implementation efforts in different service settings. This analysis will facilitate the integration of practice-based knowledge potentially not reflected in the literature.
2. A systematic integrative review of the vast and diverse literature on implementation support roles to examine our thinking through this lens, enabling the improvement of our logic. This review will build on the many studies – some of which have been highlighted in this article – that examine different aspects of the work of facilitators, knowledge brokers, consultants and others and allow for the extraction of data on e.g. the knowledge, skills, or attitudes these implementation support practitioners require, and on the contextual factors influencing their efforts.

The goal of this work is to (a) integrate this knowledge into the joint program logic, (b) describe the competencies that can be derived from this model as being potentially important for implementation support practitioners, and (c) discuss how these competencies can be developed, built and researched within real world practice settings. This last and final step will also imply to compare findings with already existing work focused on competencies as they relate to implementation science and practice. Multiple studies exist describing the curricula used with and results achieved from different types of implementation research training [6, 90, 91], while other studies have focused on identifying the knowledge, skills and attitudes needed to practice implementation or knowledge translation in real world settings [14, 16]. This literature will provide an opportunity to discuss to what degree implementation support practitioners appear to require a unique set of competencies and in which way these competencies overlap with those required by other actors involved in implementation. As part of this work, it will also be important to identify potential dilemmas and challenges faced by implementation support practitioners. The literature in general tends be optimistic about the potential of this role as a strategy to enhance implementation practice. However, studies also point to its limitations, emerging from e.g. professional boundaries, organizational norms, and a lack of authority and career pathways [92–94]. This indicates that the role itself and the institutional structures surrounding it may require further debate and development.

Hence, additional research activities will be necessary to enhance the knowledge base on implementation support practitioners, and to critically examine whether their support is adding value in practice contexts, and how this translates into positive client outcomes. First then, will it be possible to evaluate to what degree the substantial investment made in this support by local, state, and federal institutions [55] is justified. The conduct of rigorous trials comparing the effects of different implementation support practitioner reliant strategies has been – and will remain to be – an important step in this process [43, 95–97].

Additionally, rigorous qualitative studies should be prioritized to more deeply explore the mechanisms of change, together with the contextual influences affecting implementation support practitioners and their work. Relational and behavior changes build on more than a simple link between stimulus (i.e. implementation strategy use) and response (i.e. implementation behavior). It is a complex process involving the relationships, motives, identities, self-regulation, habits and rituals of individuals and groups [98]. How implementation support practitioners and their stakeholders act and respond within implementation systems will depend on the unique configuration of these factors present in implementation actors and on the interplay between them.

## Conclusion

The development of implementation support practitioners as a profession necessitates improved conceptual thinking about their role and work and how they enable the uptake and integration of evidence in real world settings. Such enhancement is possible by synthesizing the diverse literature on different implementation support roles aimed at not only consolidating what implementation support practitioners know, display, do and achieve but, importantly, also the potential mechanisms of change unfolding between their offering and the responses of their stakeholders. The logic proposed in this article will be refined as future research and practice insights deepen our understanding of the relationships between implementation support practitioners, capacity development, frontline implementation practice, and population outcomes. As such, this article introduces preliminary thinking to stimulate further research and invite input, challenge and critique from others.

## Data Availability

N/A

## Abbreviations

EBI: Evidence-Based Intervention
COM-B: Capability-Opportunity-Motivation-Behavior
U.S.: United States of America
